# A Case of Isolated Jejunal Diverticulum Presented as Free Perforation: A Rare Cause of Acute Abdomen

**DOI:** 10.7759/cureus.18809

**Published:** 2021-10-15

**Authors:** Rohik Anjum, Navin Kumar, Tanuj Singla, Rishit Mani, Bibek Karki

**Affiliations:** 1 General Surgery, All India Institute of Medical Sciences-Rishikesh, Rishikesh, IND

**Keywords:** complicated diverticulitis, bowel resection, perforation, diverticular disease, jejunal diverticulum

## Abstract

Jejunal diverticulum is a very rare disease. Diagnosis of this condition is a challenge owing to non-specific complaints of the patient. Fifteen percent cases of jejunal diverticula present with acute abdomen. Approximately 77% of small bowel diverticular disease occur with multiple diverticula. Here we describe a case of complicated isolated jejunal diverticula presenting with perforation, which was successfully treated with resection of the involved segment with anastomosis.

## Introduction

Jejunal diverticulum is a rare disease with an incidence of less than 0.5% [1]. The disease occurs more commonly in male and the incidence increases with increasing age [2]. The usual presentation of small bowel diverticular disease is non-specific, with complaints suggestive of malabsorption syndrome. Hence, diagnosis of small bowel enteropathies is usually considered. However, 15% of individuals suffering from the diverticular disease of jejunum suffer from complications like bleeding or perforation [3]. Here we describe a case of jejunal diverticulum who presented with acute abdomen and was successfully managed with surgical intervention.

## Case presentation

**Figure 1:**
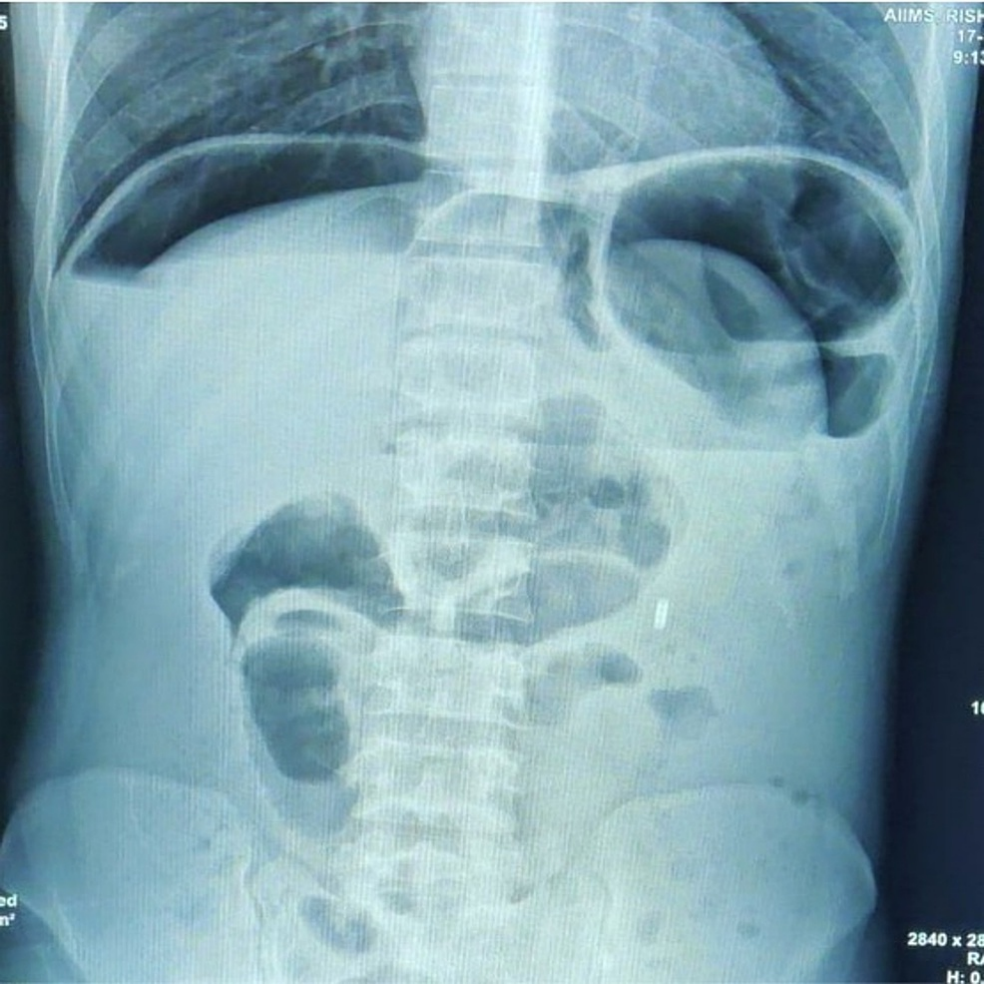
X-ray abdomen showing free air under the diaphragm

A 70-year-old gentleman, a chronic smoker, presented to theemergency with complaints of progressively increasing abdominal pain for three days and non-passage of flatus and feces for two days. He had a history of pain in the left upper abdomen on and off with altered bowel habits for the past four months; however, he did not consult any medical professional and used to take over-the-counter NSAIDs (non-steroidal anti inflammatory drugs) on and off for his pain. Vitals of the patient were as follows: pulse rate of 120 beats/min, blood pressure 120/70 mmHg, respiratory rate of 22/min, and oxygen saturation of 92% on room air. Abdominal examination was significant for distension, muscular guarding with diffuse tenderness, suggestive of peritonitis. The laboratory investigations were significant for leukocytosis with white cell count 15,100/μL, hemoglobin 13.4 g/dL, and hypoalbuminemia with albumin 2.6 g/dL. The radiograph of the abdomen revealed free air under the right dome of diaphragm (Figure 1).

**Figure 2:**
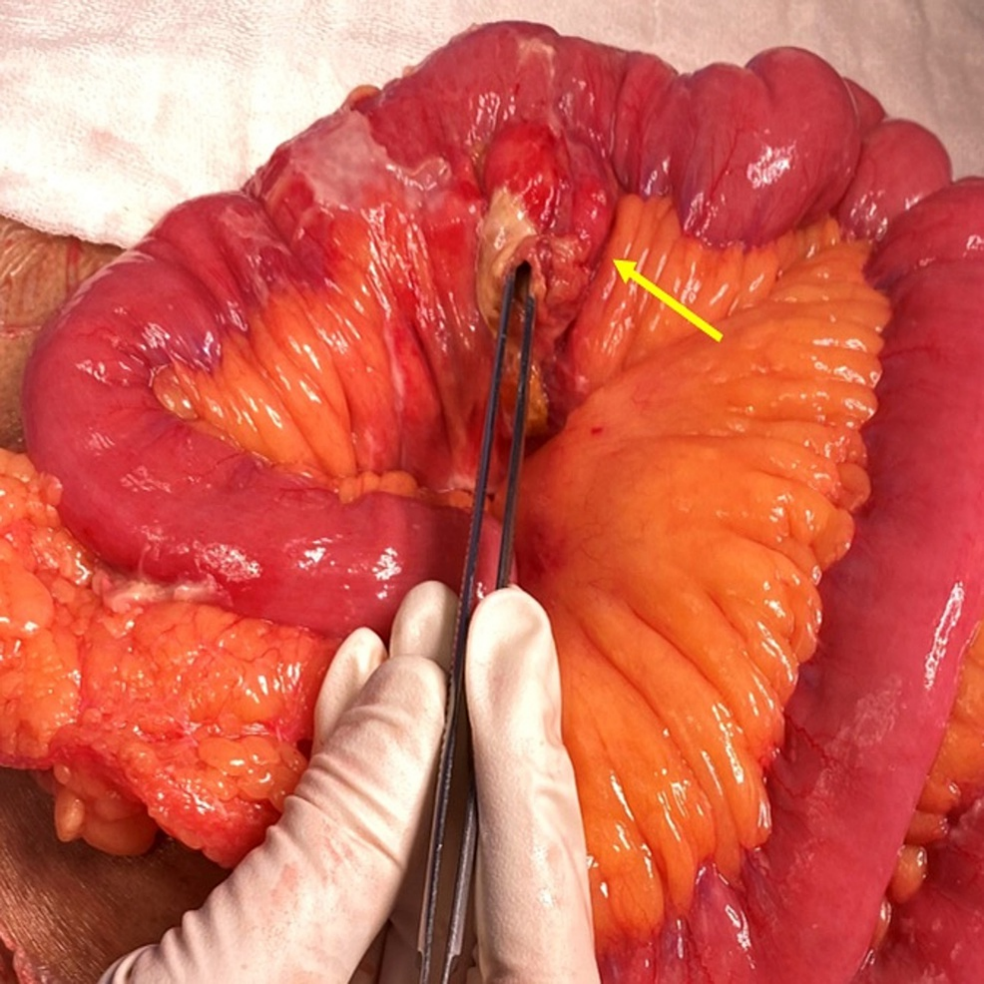
An intra-operative photograph showing the jejunal diverticulum with perforation

The presumptive diagnosis of hollow viscus perforation was made, and the patient was taken up for exploratory laparotomy. On exploration, a single diverticulum was noted 15 cm distal to duodeno-jejunal flexure and approximately 500 mL of intra-peritoneal purulent fluid was drained (Figure 2).

**Figure 3:**
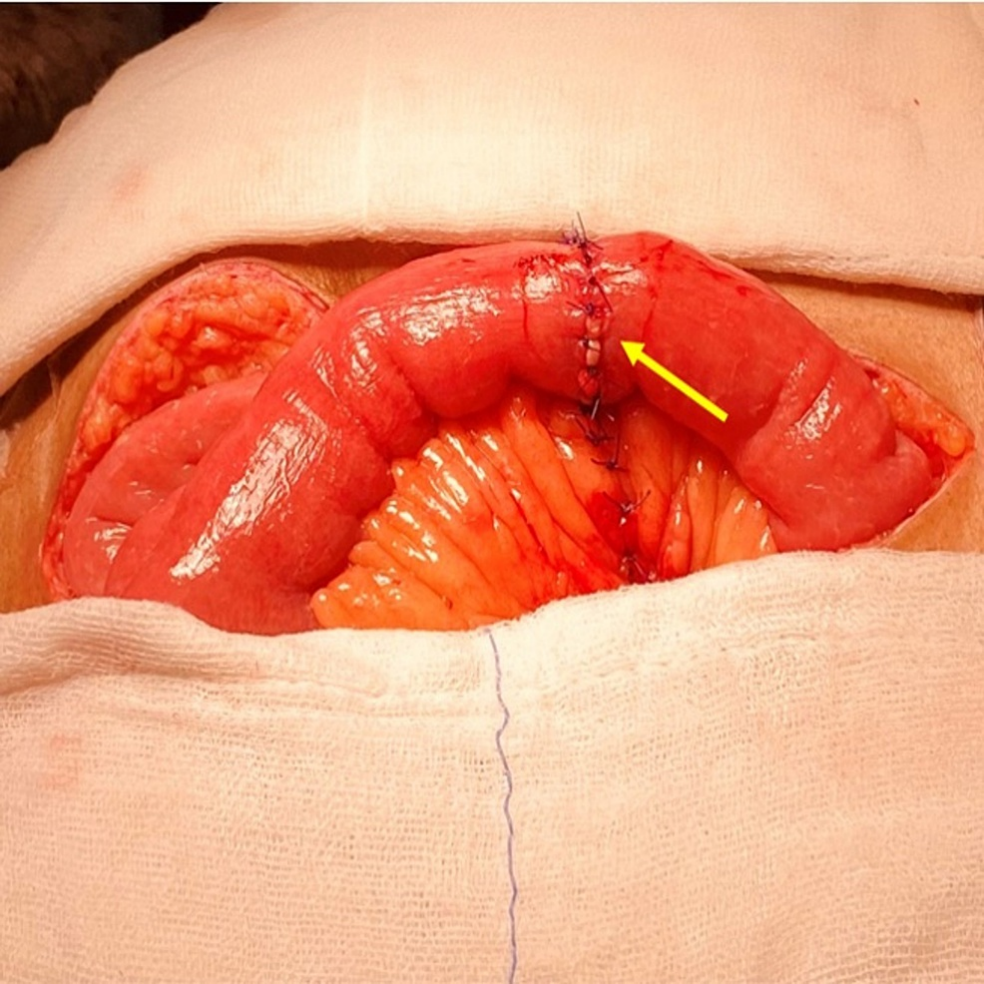
An intra-operative photograph showing the restoration of bowel continuity after the end-to-end jejunal anastomosis

No other diverticulum was identified, and the rest of the bowel was unremarkable. Resection of the perforated jejunal diverticular segment was done with adequate margins, and intestinal continuity was re-established with single-layer extra-mucosal end-to-end anastomosis of the jejunum (Figure 3).

The patient had an uncomplicated postoperative course and was allowed oral liquids on a postoperative day (POD) 1 and an oral soft diet on POD-3. He was discharged in satisfactory condition on POD-4. Patient was doing well in the follow-up period and his skin sutures were removed on POD-8. Histopathology of the resected jejunum showed perforation with acute serositis and extensive fibropurulent exudates along with inflammation in the mesentery. Granulomatous or transmural inflammation was not present in the sections studied. Screening colonoscopy was done after two weeks of discharge to rule out diverticulosis, which revealed a normal study up to the terminal ileum. Screening esophagogastroduodenoscopy was done, which showed normal study up to the second part of duodenum. He was kept on six-monthly follow-up.

## Discussion

A diverticulum refers to an outpouching that occurs from the bowel. Two types of diverticulum are described depending on the layers involved. A true diverticulum contains all the layers of the bowel, including mucosa, submucosa, muscularis, and serosa, whereas a false diverticulum (pseudodiverticulum) does not involve the muscular layer of the bowel. The Meckel's diverticulum is an example of a true diverticulum, whereas the sigmoid colon and jejunal diverticula are usually false diverticula. The pseudodiverticula contain mainly the mucosa and submucosa. Jejunal diverticulosis was first described by Somerling in 1794 and Sir Astley Cooper in 1807 [1]. Diverticulum can be single or multiple (single in our case). This was similar to the case reported by Leigh et al. in 2020 [2]. The most common site of diverticular disease in the gastro-intestinal tract is noted in the sigmoid colon [3]. The duodenal diverticula are five times more common than jejunal diverticula. However, the jejunal diverticula have a reported complication rate five times higher than the duodenal diverticula. The jejunal diverticula are 18 times more likely to perforate compared to the duodenal diverticula [4]. The most common sites of small bowel diverticular below the ligament of trietz are in proximal jejunum (75%), distal jejunum (20%), and ileum (5%) [5]. Seventy-seven percent of the cases of small bowel diverticula are multiple compared to a single isolated diverticulum [6]. Jejunal diverticula occur more frequently in males than females, usually after 40 years of age. These diverticula are generally located on the mesenteric border of the bowel, where the mesenteric vessels penetrate the jejunum [7]. Most of the cases of jejunal diverticula remain asymptomatic, with symptoms seen in only 10-30% of individuals with diverticular diseases. Complications like bleeding and intestinal perforation are seen only in 15% of individuals with diverticular diseases [8]. The risk factors for jejunal diverticula perforation include blunt abdominal trauma leading to a necrotizing inflammatory reaction in 82% of cases, foreign body impaction in 6% of cases [9], cocaine sniffing [10], NSAID usage (as noted in our case), amyloid disease and malignancy like lymphosarcoma, MEN1, and fibrous histiocytoma [11], and Ehler-Danlos syndrome [12]. Complicated diverticular disease may be diagnosed with contrast-enhanced computed tomography (CECT) scan of abdomen, which typically shows inflammatory thickening of the bowel or formation of a localized abscess or pneumoperitoneum due to perforation; however, CT enterography is a better diagnostic modality for luminal pathology [3]. The treatment for the perforated isolated jejunal diverticulum is resection of the intestinal segment, including the perforated diverticula and bowel anastomosis, as done in our case. A simple diverticulectomy is not worthwhile as it may affect the vascularity of the segment of the bowel, leading to anastomotic dehiscence and fistula formation. A non-operative trial can be attempted in stable patients or high-risk patients with localized peritonitis with IV antibiotics and CT-guided percutaneous drain insertion in cases of localized collection without diffuse peritonitis [13].

## Conclusions

Diverticular disease has to be suspected in patients presenting with unexplained malabsorption syndrome, vague abdominal pain/discomfort, or chronic anemia. Multiple jejunal diverticula are more common than single diverticulum. Complications like bleeding and perforation are seen in 15% of individuals. The treatment of complicated diverticular disease is the resection of the involved bowel segment with anastomosis. Our case was rare because the patient had a single jejunal diverticulum with free perforation. High index of suspicion is required to diagnose a small bowel diverticular perforation. On follow-up, upper and lower GI endoscopy is mandatory to rule out diverticulosis and post-operative histopathological assessment is essential to rule out small bowel malignancy.
